# Commercial use of health data—A public “trial” by citizens' jury

**DOI:** 10.1002/lrh2.10200

**Published:** 2019-08-18

**Authors:** Mary P. Tully, Lamiece Hassan, Malcolm Oswald, John Ainsworth

**Affiliations:** ^1^ Health E‐Research Centre, Division of Imaging, Informatics and Data Sciences, School of Health Sciences, Faculty of Biology, Medicine and Health University of Manchester, Manchester Academic Health Science Centre Manchester UK; ^2^ Division of Pharmacy and Optometry, School of Health Sciences, Faculty of Biology, Medicine and Health University of Manchester, Manchester Academic Health Science Centre Manchester UK; ^3^ School of Law, Faculty of Humanities University of Manchester Manchester UK; ^4^ Citizens Juries c.i.c Manchester UK

**Keywords:** commercial use, health data, public opinion

## Abstract

**Introduction:**

Surveys suggest a dichotomy in how citizens view research for public benefit and research for commercial gain. Therefore, a research initiative, such as a learning health system, which works for both public and commercial benefit, may be controversial and lower public trust.

**Methods:**

This study aimed to investigate what informed citizens considered to be appropriate uses of health data in a learning health system and why they made those decisions. Two‐paired 4‐day juries were run, with different jurors but the same purpose, expert witnesses, and facilitators. Overall, 694 people applied; 36 jurors were selected to match criteria based on demographics and privacy views. Jurors considered whether and why eight exemplars of anonymised patient data were acceptable. The exemplars were either planned initiatives to improve care pathways (Planned Examples) or possible commercial data uses (Potential Examples).

**Results:**

These citizens' juries found that all Planned and two of the Potential Examples were considered appropriate by most, but not all, jurors because they could deliver public benefit. In general, positive health outcomes for patients were more acceptable than improved efficiency of services for the NHS, although they recognised that the latter also improved health. Jurors had concerns about whether improving efficiency would lead to inequitable distribution or closure of services, based on their existing understanding from media reports. Commercial gain that accrued secondary to this benefit was acceptable, with some jurors becoming more accepting of commercial uses as they understood them better. Prioritising profit, however, was unacceptable, regardless of any governance arrangements.

**Conclusions:**

Jurors tended to be more accepting of data sharing to both private and public sectors after the jury process. Many jurors accept commercial gain if public benefit is achieved. Some were suspicious of data sharing for efficiency gains. Juries elicited more informed and nuanced judgement from citizens than surveys.

## 1 INTRODUCTION

Surveys suggest a dichotomy in how citizens view data‐intensive health research for public benefit and such research for commercial gain. About three quarters (68%‐83%) of people surveyed agree that they would be willing to share their data for health research,^1^, ^2^, ^3^ whereas only half (53%) would agree if it were commercial companies who were doing the research.^4^ Indeed, more recent figures of 39% suggest that such willingness may be decreasing.^5^

However, views on commercial use of data are more nuanced than many surveys might suggest. Qualitative research suggests the reasons why research was being conducted are very important in determining acceptability.^6^ Research that is done for public benefit is considered acceptable, regardless of the potential for commercial gain. Others have found that who profited (preferably the health service) and by how much (preferably not “obscene” amounts) influenced public opinion.^7^

Connected Health Cities (CHC) is a learning health system^8^, ^9^ in the North of England, funded by the Department of Health in England.^10^ It has three aims: (a) to improve and optimise the health and social care system to deliver better care, more efficiently, by providing actionable information to inform decision‐making at all levels, (b) to accelerate business growth in the digital health sector for the benefit of the North of England, and (c) to establish a social contract with the population that gives licence to use health care data for the public good.

To meet the first of these aims, CHC mainly uses anonymised data^11^ that have been extracted from patient records held in primary and secondary care. To meet the second, CHC works with commercial partners, providing advice and testing new technologies using anonymised and secure health data. There is a risk, however, that having *both* these aims risks lowering public trust and competing with CHC's ability to achieve its third aim.

Previous work suggests that the public are frequently unaware about how either the NHS or commercial companies use data or what governance arrangements are in place when they do.^4^, ^6^, ^7^ Therefore, deliberative methods, such as citizens' juries, are needed to ensure that members of the public have the necessary background knowledge and opportunity to weigh up the benefits and risks, before coming to considered conclusions.^12^ Surveys, where the respondents are usually asked to choose between multiple options on the assumption that they clearly understand the differences between them, would yield different, less informed, results.

Citizens' juries are based on the premise that, given enough time, opportunity, support, and resources, members of the public will make considered, informed judgements about complex matters.^13^, ^14^ The process provides an opportunity for citizens to learn about an issue and deliberate together to deliver an agreed report containing answers to the jury “mission” they have been asked to address. Using this method, initiatives using data‐intensive research can learn more about what an informed public wants and why they want it.^13^

## 2 RESEARCH AIM

This study aimed to investigate what informed citizens considered to be appropriate uses of health data in a learning health system and to explore why they made those decisions.

## 3 METHOD

Citizens Juries c.i.c., a social enterprise, were commissioned to recruit, design, and run two juries, in partnership with the Jefferson Center. The jury process followed the Center's model,^13^ and two facilitators were chosen, who were independent of CHC, including an experienced facilitator from that Centre. Two 4‐day juries were run as paired juries,^15^ with different jurors attending each jury but with the same jury mission (Box 1), expert witnesses, and facilitators. One jury was conducted in Manchester, drawing its jurors from North‐west England. The second jury (conducted 1 week later) was held in York and drew participants from North‐east England. Advice was sought from the University of Manchester Senate ethics committee, who ruled that, as the jurors were not considered research subjects and the proceedings were not being recorded, ethics committee approval was not needed.

Box 1 **The jury mission**

1. Which of the following *Planned Examples* of NHS data about patients (with identifiers like name and address removed) are acceptable?
  [choose yes, no, or unsure]
  - NHS staff working for Salford Royal Hospital get data from ambulances and hospitals. The purpose is to do research to help paramedics get better at spotting the signs of people who have had a stroke.
  - university researchers in Leeds get data from hospitals, GPs and social care about frail elderly patients. The purpose is to help GPs identify individual patients needing extra care and follow up.
  - university researchers in Liverpool get data from hospitals and GPs. The purpose is to provide information to doctors, nurses and ambulance staff about how to give more appropriate care to people suffering from alcohol‐related problems.
  - university researchers in Newcastle get data from hospital, GP and local authority records. The purpose is to plan future demand for A&E [accident and emergency] services and meet the needs of special groups (e.g., people with dementia).
2. Which of the following *Potential Examples* of NHS data about patients are acceptable?
  [choose yes, no, or unsure]
  - A pharmaceutical company requests general practice data about patients (with identifiers like name and address removed) including prescriptions, blood glucose measurements, and complications of diabetes patients. The purpose is to understand better what prescribing patterns get the best results for patients.
  - A large computer software company seeks data about patients from hospital and general practices (with identifiers like name and address removed) including patient symptoms, diagnoses and outcomes. The purpose is to enable its intelligent software to “learn” and so be used to aid future diagnosis of sepsis, a life‐threatening condition.
  - A developer of an app, designed for a wearable device like a fitbit that tracks a person's activity and measures key health indicators like blood pressure, seeks hospital data about patients (with identifiers like name and address removed). The purpose is to enable them to design the app to suggest safe fitness regimes tailored to each individual's capability and characteristics (age, weight etc.).
  - A health club chain seeks aggregated data (i.e., total numbers of patients) comparing levels of exercise, smoking history, alcohol consumption, body mass index, and blood pressure for people who have had a heart attack with those who have not had a heart attack. The purpose is to understand and identify the type of club members who are most at risk of a heart attack and monitor them.

In citizens' juries, panels are selected to be representative of the general population. Advertisements were placed in local newspapers, email circulation lists, and on an employment website. For each jury, 18 jurors and four reserves were chosen from the pool of 694 applicants in total. Applicants were asked to complete a brief questionnaire to provide information that could be used to fulfil the quotas in Table 1. A priori criteria on privacy views were used, based on one of the most recent surveys of UK opinion at the time the juries were conducted.^17^ The applicants were selected from the pool to get the closest match possible to the target quotas using these variables. If multiple applicants had identical variables, one would be picked at random with no reference to their name or address. To ensure genuine representation from all sectors of society,^15^ jurors were paid £400 plus expenses. The four reserve jurors who attended the first morning of the jury were paid £75 unless they were required as substitutes; one substitute was needed at the York jury. All jurors attended for all 4 days.

**Table 1:** A priori criteria for jury selection and demographics of actual jurors

Criteria		Jury target range	Target achieved in Manchester/York juries
Gender^a^	Women:	51% (8‐10 jurors)	9/9
	Men:	49% (8‐10 jurors)	9/9
Age range^a^	Aged 18‐29 y:	21% (2‐5 jurors)	2/4
	Aged 30‐44 y:	26% (3‐6 jurors)	6/3
	Aged 45‐59 y:	25% (3‐6 jurors)	6/6
	Aged 60 + y:	28% (3‐7 jurors)	4/5
Ethnicity^a^	White:	90/92% (15‐17 jurors)	16/15
	Non‐white	10/8% (1‐3 jurors)	2/3
Educational attainment^a^	Level 1 or no qualifications:	38/40% (5‐8 jurors)	6/6
	Level 2 or level 3 qualifications (apprenticeship & other qualifications):	37/38% (5‐8 jurors)	6/6
	Level 4 qualifications (degree level) and above:	24/23% (3‐6 jurors)	6/6
Privacy views^b^ How willing or unwilling would you be to allow your medical records to be used in a medical research study? The information given to researchers would not include your name, date of birth, address, or any contact details.			
	a) very willing	43%, 7‐8 jurors per jury	7/7
	b) fairly willing	34%, 5‐7 jurors per jury	6/6
	c) fairly unwilling d) very unwilling	c) + d): 21% (10% + 11%), 3‐4 jurors per jury	4/4
	e) do not know	3%, 0‐1 jurors per jury	1/1

a Target percentages based on UK Census.^16^

b Target percentages based on Wellcome Trust Monitor Report Wave 3.^17^

The process was planned, designed, and refined over seven months to address the jury mission (Box 1). Four planned uses of data by CHC (Examples A‐D) were selected as examples of work that CHC was planning to do. Four Potential Examples (Examples E‐H) were created based on possible projects involving commercial uses of health data that were being discussed but not actually being done.

Expert witnesses either presented factual and impartial information or partial information that argued for or against a particular viewpoint (Box 2 and Table 2). Two balancing witnesses were used, “cross‐examining” witnesses who were in favour of data sharing, on behalf of the jury members. In addition, jurors were able to question the witnesses themselves, immediately after either the presentations or cross‐examination sessions. At least 50% of the time was set aside for jury deliberations, either in small groups (which varied in membership) or as a full jury.

Box 2 **The programme of activities for both citizens' juries**

Day 1:

- Jurors complete the start‐of‐jury questionnaire
- Introduction to the event and presentation by Dr Mary Tully, followed by questions
- Group work simulation exercise (about allocation of ambulance services)
- Presentations by Dr Alan Hassey and Dr Mark Taylor, each followed by group work and discussion
- Group work on anonymising healthcare records

Day 2:

- Presentations by Prof Søren Holm and Prof John Ainsworth, each followed by group work and discussion
- ‘Cross examining’ of Prof John Ainsworth by Dr Jon Fistein on planned examples
- Group discussions on planned examples
- Jurors complete interim questionnaires on planned examples

Day 3:

- Presentations by Clare Sanderson, followed by group work and discussion
- Presentation by John McGovern
- Videos about each of the potential examples, each followed by ‘cross examining’ of John McGovern by Alexander Martin on potential examples
- Group discussions on potential examples
- Jurors complete interim questionnaires on potential examples

Day 4:

- Deliberation regarding acceptability of planned and potential examples
- Voting on acceptability of planned and potential examples
- Writing report on reasons informing jury votes
- Deliberation regarding safeguards for planned and potential examples
- Voting on safeguards for planned and potential examples
- Writing report on reasons informing decisions regarding safeguards
- Jurors complete the end‐of‐jury questionnaires

**Table 2:** Perspectives taken and information provided by impartial and partial witnesses

Witnesses	Perspective taken and information provided
Impartial witnesses:	
Dr Mary Tully, director of public engagement for connected health cities (CHC).	Information provided: Description of CHC, why the citizens' juries have been commissioned and the work that CHC will be doing over coming 2 y.
Dr Alan Hassey, a GP and former chair of the data access advisory group.	Information provided: To explain what is in a patient record and how patient records are used in the NHS for direct care and secondary uses.
Dr Mark Taylor, senior lecturer in law, University of Sheffield and chair of the confidentiality advisory group of	Information provided: The law relating to health records and including rights patients currently have with respect to their records.
Partial witnesses:	
Prof Søren Holm, professor of bioethics at the University of Manchester	Perspective: Ethical arguments for patients controlling access to patient records and ethical arguments for wider use of patient records for the benefit of the public. Information provided: Potential benefits of sharing data, problems with sharing data, and difficulties with specific informed consent models. How these conflicting interests can be reconciled. Identified ethical considerations both for patients sharing and for patients controlling patient records for uses other than direct patient care.
Prof John Ainsworth, director of CHC	Perspective: Explain four CHC planned examples of health data. Information provided: To explain how records are planned to be used within connected health cities, and why such uses are important.
Clare Sanderson, an independent consultant working for CHC and specialising in information governance	Perspective: CHC governance controls Information provided: To explain how patient records were to be used and protected by CHC.
John McGovern, chief intelligence officer of consultancy company AIMES.	Perspective: Answer questions about four CHC potential examples of health data. Information provided: To explain why private organisations seek to use health records.
Alexander Martin, journalist for The Register	Perspective: Reasons to be cautious about use of health records. Information provided: To explain the possible risks associated with commercial use of health records.
Balancing witnesses:	
Dr Jon Fistein, medical doctor and barrister	Perspective: To ask questions so that a fair balance of information is provided to jury about CHC governance controls and planned examples.
Alexander Martin, journalist for The Register	Perspective: To ask questions so that a fair balance of information is provided to jury to explain the possible risks associated with commercial use of health records in the potential examples.

Jurors completed a prejury questionnaire on the jury mission (Box 1) at the beginning of day 1 and end of day 4. This provided baseline and postjury data on their opinions on data sharing both generally and for Examples A‐H specifically. During the jury proceedings, interim questionnaires were completed to ascertain jury views on the reasons why they were leaning towards either accepting or rejecting the Examples. At the end of the jury, jurors were asked whether they had changed their mind regarding the commercial use of data. All questionnaires were completed independently and privately, using an online survey tool.

Jurors suggested reasons for and against the Jury Mission options, and the most compelling reasons chosen by voting.^12^ Each juror allocated three votes to two or three of the reasons (no reason could get all three votes). On day 4, the jury report was written by the main facilitator and jurors together, with the working document projected on a screen during discussions. The report contained the numerical data from the questionnaires, followed by an agreed statement of the strongest and most compelling reasons why the jurors had voted either for or against the acceptability of the Examples. The independent facilitator led the jurors through the report page by page to ensure that it accurately reflected their views, making any changes necessary. Afterwards, the report was sent to jury members for any final changes or comments, before the results were published online.^18^

The Jefferson Center methods to minimise bias were used.^13^ The facilitators were instructed to show no bias towards or against any of the positions being discussed and ensured that all jurors could participate equally. All main perspectives were represented among the witnesses (Table 2). Jurors completed a questionnaire at the end of each day as to whether the staff had conducted the jury in a neutral manner and, at the end of the jury, whether the facilitators had tried to influence them towards particular conclusions. The detailed jury design and all findings were published online.^18^ Additionally, all presentations by the impartial witnesses, questionnaires, and other jury materials were reviewed by an independent oversight panel. They were responsible for ensuring the integrity and fairness of the overall process, rather than any particular outcome, and requested changes to these materials where necessary to ensure this.

All questionnaire data were anonymised with a unique juror number (1‐18 for the Manchester jury and 51‐68 for the York jury), to allow tracking of opinion. The numerical data were summarised using frequencies. Qualitative data from the open questions on the questionnaires were analysed thematically and compared with the questionnaire data from the same juror. This analysis was then used to illuminate the rationale for the recommendations from the jury reports. Free‐text quotes from the questionnaires were selected to provide examples of individual juror opinions.

## 4 RESULTS

Table 3 shows the findings from the questionnaires about jurors' attitudes to sharing data generally, showing how jurors changed their minds between the beginning and end of the jury. Tables 4 and 5 show whether the jurors found the Planned and Potential Examples to be acceptable pre and postjury. All Planned and two of the Potential Examples were considered appropriate by most, but not all, jurors. Tables 6 and 7 show the strongest and most compelling reasons that the jurors selected for inclusion in the report, with the numbers of votes. The main themes from the qualitative data analysis are described below.

**Table 3:** Changes in privacy views from recruitment to end of jury

	Manchester				York			
Privacy views	Prejury (n)	Changed to:	Postjury (n)	Changed from:	Prejury (n)	Changed to:	Postjury (n)	Changed from:
a) Very willing	7	3 a ‐> a 4 a ‐> b (−)	8	3 a ‐> a 4 b ‐> a (+) 1 e ‐> a (+)	7	6 a ‐> a 1 a ‐> b (−)	11	6 a ‐> a 4 b ‐> a (+) 1 e ‐> a (+)
b) Fairly willing	6	2 b ‐> b 4 b ‐> a (+)	10	4 a ‐> b (−) 2 b ‐> b 2 c ‐> b (+) 2 d ‐> b (+)	6	2 b ‐> b 4 b ‐> a (+)	4	1 a ‐> b (−) 2 b ‐> b 1 c ‐> b (+)
c) Fairly unwilling	2	2 c ‐> b (+)	0		2	1 c ‐> b (+) 1 c ‐> e	1	1 d ‐> c (+)
d) Very unwilling	2	2 d ‐> b (+)	0		2	1 d ‐> c (+) 1 d ‐> d	1	1 d ‐> d
e) Do not know	1	1 e ‐> a (+)	0		1	1 e ‐> a (+)	1	1 c ‐> e

Key: + = became more willing; − = became less willing.

**Table 4:** Results from pre‐ and postjury questionnaires as to whether planned examples A to D were acceptable (see Box 1 for details), completed individually by jurors, including changes in opinions

	Manchester				York			
Planned examples^a^	Prejury (n)	Changed to:	Postjury (n)	Changed from:	Prejury (n)	Changed to:	Postjury (n)	Changed from:
Example A (stroke)								
Yes, acceptable	15	14 Y ‐> Y 1 Y ‐> U	17	14 Y ‐> Y 2 U ‐> Y 1 N ‐> Y	15	15 Y ‐> Y	18	15 Y ‐> Y 2 U ‐> Y 1 N ‐> Y
Unsure	2	2 U ‐> Y	1	1 Y ‐> U	2	2 U ‐> Y	0	
No, not acceptable	1	1 N ‐> Y	0		1	1 N ‐> Y	0	
Example B (frailty)								
Yes, acceptable	16	8 Y ‐> Y 2 Y ‐> U 6 Y ‐> N	9	8 Y ‐> Y 1 U ‐> Y	14	11 Y ‐> Y 3 Y ‐> U	13	11 Y ‐> Y 2 N ‐> Y
Unsure	2	1 U ‐> Y 1 U ‐> U	3	1 U ‐> U 2 Y ‐> U	1	1 U ‐> N	3	3 Y ‐> U
No, not acceptable	0		6	6 Y ‐> N	3	2 N ‐> Y 1 N ‐> N	2	1 U ‐> N 1 N ‐> N
Example C (alcoholism)								
Yes, acceptable	12	9 Y ‐> Y 1 Y ‐> U 2 Y ‐> N	13	9 Y ‐> Y 3 U ‐> Y 1 N ‐> Y	11	11 Y ‐> Y	16	11 Y ‐> Y 4 U ‐> Y 1 N ‐> Y
Unsure	5	3 U ‐> Y 1 U ‐> U 1 U ‐> N	2	1 Y ‐> U 1 U ‐> U	5	4 U ‐> Y 1 U ‐> U	1	1 U ‐> U
No, not acceptable	1	1 N ‐> Y	3	2 Y ‐> N 1 U ‐> N	2	1 N ‐> Y 1 N ‐> N	1	1 N ‐> N
Example D (A&E demand)								
Yes, acceptable	13	9 Y ‐> Y 4 Y ‐> N	10	9 Y ‐> Y 1 U ‐> Y	13	11 Y ‐> Y 1 Y ‐> U 1 Y ‐> N	13	11 Y ‐> Y 2 U ‐> Y
Unsure	4	1 U ‐> Y 3 U ‐> U	3	3 U ‐> U	2	2 U ‐> Y	1	1 Y ‐> U
No, not acceptable	1	1 N ‐> N	5	4 Y ‐> N 1 N ‐> N	3	3 N ‐> N	4	1 Y ‐> N 3 N ‐> N

a See Box 1 for details of Examples A to D.

**Table 5:** Results from pre‐ and postjury questionnaires as to whether potential examples E to H were acceptable (see Box 1 for details), completed individually by jurors, including changes in opinions

	Manchester				York			
Planned examples^a^	Prejury (n)	Changed to:	Postjury (n)	Changed from:	Prejury (n)	Changed to:	Postjury (n)	Changed from:
Example E (pharma)								
Yes, acceptable	8	7 Y ‐> Y 1 Y ‐> N	13	7 Y ‐> Y 3 U ‐> Y 3 N ‐> Y	10	10 Y ‐> Y	14	10 Y ‐> Y 2 U ‐> Y 2 N ‐> Y
Unsure	5	3 U ‐> Y 2 U ‐> N	1	1 N ‐> U	3	2 U ‐> Y 1 U ‐> N	0	
No, not acceptable	5	3 N ‐> Y 1 N ‐> U 1 N ‐> N	4	1 Y ‐> N 2 U ‐> N 1 N ‐> N	5	2 N ‐> Y 3 N ‐> N	4	1 U ‐> N 3 N ‐> N
Example F (sepsis software)								
Yes, acceptable	8	7 Y ‐> Y 1 Y ‐> U	15	7 Y ‐> Y 5 U ‐> Y 3 N ‐> Y	5	5 Y ‐> Y	15	5 Y ‐> Y 7 U ‐> Y 3 N ‐> Y
Unsure	5	5 U ‐> Y	2	1 Y ‐> U 1 N ‐> U	9	7 U ‐> Y 1 U ‐> U 1 U ‐> N	1	1 U ‐> U
No, not acceptable	5	3 N ‐> Y 1 N ‐> U 1 N ‐> N	1	1 N ‐> N	4	3 N ‐> Y 1 N ‐> N	2	1 U ‐> N 1 N ‐> N
Example G (fitness app)								
Yes, acceptable	2	2 Y ‐> N	1	1 U ‐> Y	5	1 Y ‐> Y 2 Y ‐> U 2 Y ‐> N	1	1 Y ‐> Y
Unsure	8	1 U ‐> Y 7 U ‐> N	1	1 N ‐> U	5	2 U ‐> U 3 U ‐> N	4	2 Y ‐> U 2 U ‐> U
No, not acceptable	8	1 N ‐> U 7 N ‐> N	16	2 Y ‐> N 7 U ‐> N 7 N ‐> N	8	8 N ‐> N	13	2 Y ‐> N 3 U ‐> N 8 N ‐> N
Example H (health club)								
Yes, acceptable	5	5 Y ‐> N	0		4	2 Y ‐> U 2 Y ‐> N	0	
Unsure	4	4 U ‐> N	0		4	1 U ‐> U 3 U ‐> N	5	2 Y ‐> U 1 U ‐> U 2 N ‐> U
No, not acceptable	9	9 N ‐> N	18	5 Y ‐> N 4 U ‐> N 9 N ‐> N	10	8 N ‐> N 2 N ‐> U	13	2 Y ‐> N 3 U ‐> N 8 N ‐> N

See Box 1 for details of Examples E to H.

**Table 6:** The strongest, most compelling reasons that highlight the potential benefits or potential drawbacks of the planned examples of anonymised data

Potential benefits:	These planned examples:
Manchester jury	• May lead to improved treatments, services, and care delivery and eventually to better health outcomes and more lives saved (24 votes) • Could strengthen research and help identify health trends, areas of concentrated positive or negative health conditions (“hot spots”), and special populations who are affected by different conditions or who have better than average health outcomes (15 votes)
York jury	• May lead to better diagnoses of conditions, more effective treatments, and improved health outcomes for patients (26 votes) • Might allow NHS to more efficiently target the use of resources for particular conditions or communities, which could allow more effective use of funds and resources (14 votes) • Could lead to patients being better informed about their healthcare options which would give them more control over their care and choice in medical decisions (11 votes)
Potential drawbacks:	
Manchester jury	• May generate findings or research conclusions that are not supported with funding commitments so they may not lead to implementation (13 votes) • May lead to an increase in geographic, community‐based, and social stereotyping and stigmatisation as well as inequitable distribution of resources (“postal code lottery”) (11 votes)
York jury	• Do not guarantee that general public will be aware of or support the use of their anonymised records for these purposes (12 votes) • Create the possibility for data breaches among partner organisations, especially in cases where medical and nonmedical (social care) records are linked (12 votes) • Do not have guaranteed or committed funding for implementation so might end up being abandoned and wasting resources (7 votes)

**Table 7:** The strongest, most compelling reasons that highlight the potential benefits or potential drawbacks of the Potential Examples of anonymised data

Potential benefits:	These potential examples:
Manchester jury	• May expedite research and development of new drugs, products, and services, which could lead to decreased costs and improved services for consumers (17 votes) • May help identify gaps that exist in health services, technologies, and drugs, which could improve care outcomes, improve well‐being, and, ultimately, save lives (17 votes)
York jury	• Could lead to the development of efficient and cost‐effective drugs, treatments, and diagnosis programmed that might lower costs for NHS and patients (25 votes) • Might allow health professionals to recognise conditions earlier and improve the treatment of some conditions (15 votes) • Could lead to the development of technologies or approaches for one condition that might be beneficial to others, creating a spill‐over effect (12 votes)
Potential drawbacks:	
Manchester jury	• May not satisfactorily demonstrate that the goal for data usage is public benefit as opposed to simple commercial gain or profit for a company (25 votes) • Do not always satisfy concerns about proper safeguards and data protection practices by private companies and other commercial interests (10 votes)
York jury	• Tend to be driven primarily by the need to increase or generate profit without ensuring a clear public benefit from the use of people's personal health data (25 votes) • Can increase reliance on technology for identifying and diagnosing illness, leading to less clinical expertise for medical professionals and limiting the patient/doctor relationship (12 votes) • Could lead to the development of products that are not used by individuals or communities, who might need them most due to price or other accessibility obstacles (7 votes)

## 5 PUBLIC BENEFITS

A key factor for the jurors to find the Examples acceptable was that it delivered public benefit. In the Manchester jury report, the potential benefits of acceptable Planned Examples, were that they had “the potential of benefitting the public through improving care and saving lives and are likely to have multiple benefits or lead to a ripple effect where one finding may generate other improvements later.” Similarly, for the Potential Examples, the juries highlighted that one of the compelling reasons for finding them acceptable was if “both a private and a public benefit can be adequately demonstrated” (Manchester jury report).

Examples of public benefit included saving lives (as with Examples A and F) and improvements in drugs, treatments, and other health care services (as with Examples B‐E), particularly where such improvements could lower costs or provide other direct benefits to the NHS. This was seen in the increase in the number of jurors, who found Example E acceptable between the pre and postjury questionnaires (Table 5). Although some jurors were concerned about who would profit from the use of data in Example E, achieving “the greater good” (Juror 63) resulted in most jurors considering it an acceptable use. Even one of the jurors who remained very unwilling to share data throughout the process wrote that Example A was “a clear potential lifesaver” (Juror 60).

## 6 NHS BENEFITS

The York jury highlighted in their report that the Examples should “demonstrate the potential for more targeted use of resources and possible cost savings for the NHS and for residents.” Other reasons why the Examples were acceptable highlighted the role that data could play in expediting and advancing research, possibly leading to decreased costs in the future. Both juries mentioned that these Planned Examples could have both long‐term and unforeseen benefits, suggesting that they accepted that the outcome of such initiatives might not necessarily be known in advance.

However, particular concerns were included in both reports about how the findings from the Examples might not be followed up by an NHS commitment or financial support to follow through and deliver any new services. In one case (Example D), jurors did not understand “how can you plan future demand for accidents and emergencies as the whole purpose for this department is that it is for the unanticipated” (Juror 55). There were also some negative comments about how the proposed work might benefit staff rather than patients. A number of jurors, who had initially been positive about Example D in the prejury questionnaires, became negative after the deliberations, because they did not see how it was possible to practically achieve what they believed was being proposed. In addition, there were concerns about the perceived drive for efficiency, with Juror 7 writing about seeing the “continuous closure of A&E [accident and emergency departments] in the North of England” in the media. Some jurors, therefore, saw example D, as a waste of resources or a way for “government” to justify closing of services.

Although targeted use of NHS resources was seen as important generally (Table 6), when it came to discussion of Planned Examples that actioned this (Examples B and C), they were not necessarily found acceptable by some jurors. Example B was found to be less acceptable postjury than before the jury deliberations. Much of the discussion was about the practicalities of delivering the new service to frail elderly people, rather than about the uses of health data. Issues about the “sensitivity” and “pride” of the people with frailty and “intrusiveness” of delivering the service changed the mind of one person (Juror 13). Whether the NHS could or would deliver this new service within current resources was hotly debated. In the interim questionnaire, Juror 2 stated, “there are already too few resources and no money. Is it a pointless exercise?”

## 7 COMMERCIAL BENEFITS

Eleven Manchester and 10 York jurors reported they had changed their minds about the use of data by commercial companies and explained their rationale. From their free‐text answers, those jurors who had changed their mind became more positive whilst those who maintained their original view remained quite negative about such data uses. No one reported changing from being positive to being negative about commercial use of data. A common reason given for jurors changing their mind was that they felt more informed, particularly about safeguards. Some jurors described how they had not really understood the issue before and that they had been convinced by the amount of safeguards that were in place for commercial use. One of the Manchester jurors wrote, “My default setting at first was to disregard the sharing of my data or its use from there on in. I now can make more informed choices and have become far more open minded and less rigid in my thinking.” (Juror 18). Another from York stated that “balance of the partnership to be weighted in favour of greater public good, with spin offs for the company, and not the other way round.” (Juror 68).

Where the NHS was closely involved, commercial involvement was seen as more acceptable. The Manchester jury even highlighted in their report the possibility of “commercial organisations behaving in a more ethical manner because they accept and adopt the NHS principles”. One juror was initially very sceptical about Example E, stating that the company was “driven by profit not the general well‐being of the patient” (Juror 68) in the interim questionnaire. However, discussion of the safeguards that would be put in place convinced the juror to find this case acceptable in the postjury questionnaire. However, for many jurors, there remained a feeling of distrust as to “the true plans of commercial companies” (Juror 10), particularly if its use would “just be for financial gain” (Juror 6) rather than to “benefit the public as a whole” (Juror 12).

In their report, the York jury stated “We are deeply concerned about using patient data for reasons which prioritise generating profit for private organisations over public benefit,” a sentiment that was echoed in the Manchester report, with concern over prioritising “commercial gain (financial, reputational)”. Commercial gain was expected to be offset by significant public benefits and the Potential Examples where these public benefits were not clear to the jurors were considered unacceptable.

Many of the jurors who were uncomfortable with commercial access did not trust the companies' motives. For example, some jurors were concerned regarding Example F, which they saw as allowing algorithms to replace the health care professional and patient interaction that could be harmful in the longer term. Those jurors who were initially unwilling to share their data were quite distrustful of the pharmaceutical company's “agenda” in Example E, and there were comments about the “balance too far towards company versus patient” (Juror 2).

There were also concerns as to the ability and willingness of commercial companies to protect the data appropriately, therefore any “benefits I have now considered still don't outweigh a possible data breach in my opinion” (Juror 60).

For both Examples G and H, jurors generally started off unsure as to their acceptability and finally finding them unacceptable. For most jurors, the reason given was that there was “more commercial than individual benefit” (Juror 60). Even those people who were positive about Examples E and F found these two Examples unacceptable. Working with the NHS was seen as being a way that companies could differentiate themselves from others, but this was seen as a commercial benefit and therefore unacceptable.

“These uses are unacceptable, no matter how minimal the risk of breach, the data will be used unprofessionally, by unqualified people who have just been given some data. Prejudice and stigmas will increase. Facts can be manipulated or used out of context, to give companies the outcomes they require. … Also these cannot be regulated, therefore the trust factor is too high a risk.” (Juror 55).

## 8 POTENTIAL FOR BIAS

Table 8 shows of the responses to the final jury questions about whether anyone had tried to influence their opinion. Few pertained to bias by the facilitators. Comments were given by a few of the jurors, who perceived such influence or bias. Several jurors commented that the question and answer session between the balancing witnesses and the partial witnesses seemed “rehearsed,” particularly in the second jury (when the session was being run for the second time). Another juror, who remained sceptical about commercial use throughout the jury process, commented on the perceived impact of how the sessions were ordered:

**Table 8:** Responses to jury questionnaire about whether anyone had tried to influence their conclusions

	Manchester			York		
	Facilitators	Impartial witnesses	Someone else	Facilitators	Impartial witnesses	Someone else
Not at all	17	15	15	15	16	16
Perhaps occasionally	0	2	2	2	1	1
Sometimes	1	1	1	0	1	1
Often	0	0	0	0	0	0
Very often	0	0	0	0	0	0

“The whole process has been organised in such a way that has made me feel that CHC has already made their mind up and the real question is potential uses which was presented to us at the end. By this time most people were tired … made people question the uses less than if these uses were presented at the beginning of the jury.” (Juror 55).

One juror also commented about how the other jurors had changed their mind as a result of the deliberation process:

“As I learned more from the other jurors during our discussions some of my opinions were changed or modified.” (Juror 65).

In summary, the key issues presented by the jurors were that saving lives and providing unarguable patient benefits, such as Examples A and F, were almost universally acceptable. However, jurors were worried about whether the NHS would be able to deliver on service improvements such as Example C, given the rhetoric they heard regularly in the media about the NHS being under‐resourced. Overall, NHS benefit was not seen as being as important as direct patient impact. Some, but not all, jurors became more accepting of commercial uses as they understood them, and the associated governance, better. Commercial gain that occurred secondary to achieving public benefit was generally accepted. Commercial uses that prioritised generating profit and did not produce health benefits for the public were unacceptable, regardless of any safeguards for the data.

## 9 DISCUSSION

This study has provided further insight into the views of citizens about the commercial use of data. When engaging in a deliberative process, citizens do not universally veto commercial use of data but apply the same “public benefit criterion” that they do to other data uses. Some citizens accepted certain uses, and some changed their minds. This study provides an insight into that deliberative process and the principles behind the reasoning as applied to commercial uses.

This study has highlighted that citizens' juries elicit a more informed judgement from citizens than do surveys, and this can add legitimacy to decision‐making about both the work of a learning health system and the associated public engagement. Many of the jurors changed their mind to become more accepting of data uses as they understood more about them, as we have found before using this technique.^12^ Citizens' juries are arguably a way of providing “informational transparency” and “participatory transparency” in public engagement about data‐intensive health research.^19^ It has been suggested that citizens' juries *symbolically* represent the community,^20^, ^21^ although they are not usually given public accountability for their decisions. Citizens' juries are complex and relatively costly. Hence, they are best suited to situations where there is a need to involve the public in decision‐making about relatively complex matters, when time can be given to deliberation in order to reach an informed decision, and where the decision‐making body is willing to consider the jury recommendations.^15^

The finding that citizens value data‐intensive health research for public benefit is not new and could be considered the key condition for acceptability by the public.^4^, ^6^, ^12^ It has been described using a number of terms, such as public good, societal responsibility, or even altruism.^2^, ^22^, ^23^, ^24^, ^25^ In one survey, for example, 67% agreed that their health data were of value “to help improve things for people other than me,” and only 12% disagreed.^4^ “Provable and sufficient public benefit” was the first test that people applied to decide whether commercial uses of data were acceptable.^4^ Only when this test was passed were other factors (such as who was doing the work or what safeguards were in place) taken into account. We saw similar behaviour with our jurors when discussing Examples G and H. After the deliberations, these were not seen as having sufficient public benefit and were therefore considered unacceptable, regardless of any safeguards in place for the data (such as aggregation in Example H).

In our jury reports, public benefit was described as “improving care and saving lives,” and ongoing discussions suggested that improving service efficiency was NHS and *not* public benefit. Despite how frequently the term is used in the literature, there is no agreed definition of what public benefit it means in the context of data‐intensive health research. Public sector professionals (including health care professionals) found it difficult to clearly articulate what was the public benefit from the effective use of data.^26^ However, three things eventually emerged: providing societal benefits through better public services, delivering improved outcomes for communities, and enabling research. Elsewhere, members of the public acknowledged value in keeping public benefit only loosely defined, beyond the general concept that research should benefit society as widely as possible.^27^ Research to improve service efficiency, thus, could be considered as a public benefit. Our study suggests that, when considering potential uses of health data by commercial organisations, this public benefit must be made explicit; otherwise, citizens will not find the use acceptable.

There is heterogeneity in public opinions, with different “mind sets” and “publics” described in the literature.^4^, ^28^ There were a few jurors, particularly at York, who were adamant that commercial uses of data were never acceptable. Other research has found that 17% of the general public would not accept commercial use of data at all,^4^ and qualitative studies found that there is a belief in a hidden agenda with commercial companies.^19^ These negative opinions can become more positive if people are involved in deliberative processes, such as focus groups^4^ or citizens' juries.^12^ However, even at the end of our jury process, some jurors still felt the same way. Others have found that deliberation and debate can strengthen, rather than change, opinions.^29^ This raises the question as to whether, no matter how open commercial companies were as to what they were doing, they would not be trusted by some.

Our findings also highlight the importance of taking into account the knowledge and experience that members of the public already have. When broadening the conceptualisation of public trust in health care, Gille and colleagues have argued for the importance of including personal experience of services and the influence of mass media in the origin of public trust.^30^ We saw how jurors' prior beliefs about how the NHS operates resulted in them stating concerns about whether improving efficiency would inevitably lead to inequitable distribution or closure of services and whether the lack of funding or political will to implement new services would lead to increased public dissatisfaction because of expectations having been falsely raised. This suggests the importance, again, of being explicit in public engagement about how existing services are delivered before explaining how data will be used to improve those services in a learning health system.

A key strength of this study was that we ran the juries according to the “ideal” suggested in a recent systematic review.^15^ We had a member of Jefferson Center staff act as lead jury facilitator, ensuring that we adhered to their model.^12^, ^13^ This ensured that the citizens' juries demonstrated the three important characteristics of deliberative democracy: inclusivity, deliberation, and active citizenship,^15^ whilst minimising bias. Having jurors with a range of views on data privacy that mirrored the general population, because of our selection criteria (Table 1), ensured that the deliberation process challenged all jurors to think broadly on the topics.

Bias, both conscious and unconscious, is an important criticism of citizens' juries.^31^ Almost every design choice, even down to a bullet point on a presenter's slide or the order information is presented, could be challenged on grounds that it might manipulate the citizens' jury towards one outcome or another. Bias can thus be monitored and minimised but not eliminated. In our case, for example, the decision to use balancing witnesses were seen as a potential bias by some jurors during the second jury in York, when the questioning was seen as pre‐prepared. This was an inevitable consequence of the same witnesses having already been questioned in a similar fashion the week before in Manchester.

Citizens' juries inevitably involve small numbers of people and are designed to deliver the decision of a minipublic, rather than the individuals within it. Thus, they should not be used to compare opinions of minority groups within the jury—other qualitative methods such as focus groups would be more appropriate for such a task. Another limitation of the study was the evidence that even with the presentations by the witnesses and the opportunity to ask questions, some jurors still did not understand the way in which the example cases could be taken up and put into practice by the NHS. The need to avoid bias in the jury process meant that there was little opportunity to correct misunderstandings. This raises questions as to whether an improvement for future citizens' jury work might be the presence of an independent expert throughout that jurors could call upon for factual information at any time, in the same way as is done in deliberative focus groups.^32^ This has been used before with citizens' juries but with mixed results, and it can itself be a source of bias.^13^

## 10 CONCLUSIONS

These citizens' juries found that all Planned and two of the Potential Examples were considered appropriate by most, but not all, jurors because they could deliver public benefit. Commercial gain that accrued secondary to this benefit was acceptable, with some jurors becoming more accepting of commercial uses as they understood them better. Prioritising profit, however, was unacceptable, regardless of any governance arrangements. Positive health outcomes for patients were more acceptable than improved efficiency of services for the NHS. Jurors had concerns about whether improving efficiency would lead to inequitable distribution or closure of services, based on their existing understanding from media reports. Data‐use initiatives like learning health systems need to ensure that they understand public views and opinions about the improvements that they intend to make, so that they can plan their public engagement accordingly.

## CONFLICT OF INTEREST

M.O. is Director of Citizens Juries c.i.c. (Community Interest Company), a social enterprise dedicated to designing and running citizens' juries, and was commissioned to deliver these juries. The other authors state that they have no conflicts of interest to declare.
